# The Trypanosomatid Pr77-hallmark contains a downstream core promoter element essential for transcription activity of the *Trypanosoma cruzi* L1Tc retrotransposon

**DOI:** 10.1186/s12864-016-2427-6

**Published:** 2016-02-09

**Authors:** Francisco Macías, Manuel Carlos López, M. Carmen Thomas

**Affiliations:** Instituto de Parasitología y Biomedicina López-Neyra, Consejo Superior de Investigaciones Científicas (IPBLN-CSIC), PTS Granada, Avda. del Conocimiento S/N, 18016 Granada, Spain

**Keywords:** Trypanosomatids, *Trypanosoma cruzi*, Transcription, Promoter, Downstream promoter element, L1Tc

## Abstract

**Background:**

Trypanosomatid genomes are highly colonized by non-LTR retroelements that make up to 5 % of the nuclear genome. These elements are mainly accumulated in the strand switch regions (SSRs) where polycistronic transcription is initiated and have a 77 nt-long sequence - Pr77 - at their 5′ ends. L1Tc is the best represented retrotransposon in the *Trypanosoma cruzi* genome and is a potentially functional autonomous element that encodes its own retrotransposition machinery. The Pr77 of the *T. cruzi* L1Tc element activates gene transcription via RNA polymerase II, generating abundant, unspliced transcripts which are translated.

**Results:**

The present manuscript describes the identification of a downstream core promoter element (DPE) in the L1Tc Pr77 sequence. Just four nucleotides long (CGTG), it covers in Pr77 positions +25 to +28 of the described L1Tc transcription start site. The Pr77-DPE motif is conserved in terms of sequence composition and position in the Pr77 of most trypanosomatid non-LTR retrotransposons, independent of the coding or non-coding capacity of these retroelements. Transcription assays in *T. cruzi* stable transfectants with vector containing point mutations at 17 locations of the Pr77 nucleotide sequence evidence that the DPE motif is essential for the promoter function of Pr77. Furthermore, the obtained data show that other nucleotides also contributed to the promoter function of Pr77. In addition, the presented results indicate that parasite nuclear proteins specifically bind to different regions of the Pr77 sequence although the strongest binding is to the DPE motif. Moreover, it is shown that the DPE sense single-stranded sequence is being required in DNA-protein recognition of nuclear factors.

**Conclusions:**

The Pr77 sequence present in most of non-LTR retrotransposons of trypanosomatids contains a downstream core promoter element (DPE) which is conserved in terms of nucleotide composition and location. The Pr77-DPE motif is essential for the transcriptional activity of Pr77 although other nucleotides are also involved. DPE has a high affinity binding for nuclear proteins in *T. cruzi*. The wide retroelement-mediated distribution of Pr77 suggests that it may represent an important tool for regulating gene expression in trypanosomatids.

**Electronic supplementary material:**

The online version of this article (doi:10.1186/s12864-016-2427-6) contains supplementary material, which is available to authorized users.

## Background

*Trypanosoma cruzi* is the etiological agent of Chagas disease, a chronic sickness that affects 7 million people worldwide, mostly in Latin America where it was entirely confined although it has now spread to other continents (http://www.who.int/mediacentre/factsheets/fs340/en/). This intracellular, protozoan parasite has been extensively studied because of its impact on human health, but also because of the interesting molecular characteristics of the family *Trypanosomatidae*, which shows some unusual features of gene transcription.

The sequencing of trypanosomatid genomes has shown them all to contain a large number of active and inactive retrotransposons that together make up to 5 % of the nuclear genome [1]. Retrotransposons are mobile genetic elements that move from one site in the genome to another via the reverse transcription of their own RNA. The best represented retrotransposons in trypanosomatids are the non-LTR retrotransposons, which move via a mechanism known as target-primed reverse transcription (TPRT) [2], in which the RNA encoded by the element is reverse transcribed and the newly synthesized DNA copy inserted at a new site in the genome [3]. Among the non-LTR retrotransposons are the long interspersed nucleotide elements (LINEs), i.e., long, autonomous elements that code for proteins that mediate the transposition mechanism, and the short interspersed nucleotide elements (SINEs), i.e., short non-coding elements that have to be mobilized *in trans* by the enzymatic machinery encoded by LINEs. The best characterized LINE is L1Tc of *T. cruzi* [4, 5] which, together with the *ingi* element of *T. brucei* (Tb*ingi*) were the first identified elements in, and the name-givers to, the *ingi*/L1Tc clade [6]. Homologous elements are now known in the *T. vivax* (*Tvingi*) and *T. congolense* (L1Tco and Tco*ingi*) genomes. Truncated versions of these LINEs - short, non-coding elements - also exist in the *T. cruzi, T. brucei*, *T. vivax* and *T. congolense* genomes (NARTc, TbRIME, TvRIME and TcoRIME, respectively) [7, 8]. Further, trypanosomatid genomes contain versions of the long and short elements known as degenerate *ingi*/L1Tc-related elements (DIRES) [6] and short interspersed degenerate retrotransposons (SIDERs). These have accumulated a huge number of mutations that have disabled their coding capacity. The expansion of the SIDERs has been different in different genera. Thus, in the *T. brucei* genome, some 20 copies of SIDERs have been detected per haploid genome, whereas in *Leishmania* species there are around 2000 copies per haploid genome [9].

All these retrotransposons share a common 77 nt sequence at their 5′ ends known as Pr77, the Pr77-signature, or the Pr77-hallmark, independent of their length, their autonomy in terms of mobility related to their coding/non-coding capacity, and their degree of degeneration [7, 8, 10]. Pr77 was originally described in L1Tc and was shown to activate the transcription of downstream genes, generating abundant transcripts via RNA polymerase II [11]. Pr77 promoter–derived unspliced transcripts initiate at or close to nucleotide +1 and are efficiently translated [11]. L1Tc has coding capacity for an apurinic/apyrimidinic (AP) endonuclease (with 3′ phosphatase and 3′ phosphodiesterase activities [12–14], a reverse transcriptase [15], RNaseH [16], and a nucleic acid chaperone [17, 18]. It also codes for an *in vitro-* and *in vivo*-active, self-cleaving 2A sequence (L1Tc2A) that may well determine the composition and proportions of the products translated from L1Tc [19]. The recent identification of a functional hepatitis delta virus-like structure covering the first 77 nt of the 5′ end of the L1Tc and NARTc retrotransposon mRNAs-L1TcRz, a structure with HDV-ribozyme function [20, 21], is the first dual promoter/ribozyme system to be discovered that works at the DNA/RNA levels respectively. The Pr77 signature-bearing retrotransposons may well be responsible for the expansion of these functions across trypanosomatid genomes [22].

The organization of trypanosomatid genomes is unusual. The genes are tandemly repeated and organized in long clusters that converge or diverge in the strand switch regions (SSRs) where polycistronic transcription initiates [23–25]. Since the SSRs appear to be accumulation regions for non-LTR retrotransposon sites, it has been suggested that Pr77 promoter activity may be responsible for the transcription of adjacent genes and polycistrons [11].

This paper describes the identification, in Pr77 of L1Tc, of a downstream core promoter element (DPE) motif. This 4 nt-long motif (CGTG) is located at position +25 to +28 relative to the +1 transcription start site (TSS) of L1Tc mRNA. The composition and location of the DPE motif was found to be conserved in most Pr77 signatures from trypanosomatid retrotransposons. Overexpression of a reporter gene by Pr77 in *T. cruzi* transfectants, along with point mutations introduced into Pr77, showed the DPE to be involved in Pr77 promoter activity, although other nucleotides in this sequence were found to be also important in transcription activation. Furthermore, it is also shown that parasite’ nuclear factors specifically bind to the DPE sense sequence and other positions in Pr77 sequence.

## Results

### Pr77 promoter contains a downstream promoter element

**Fig. 1:**
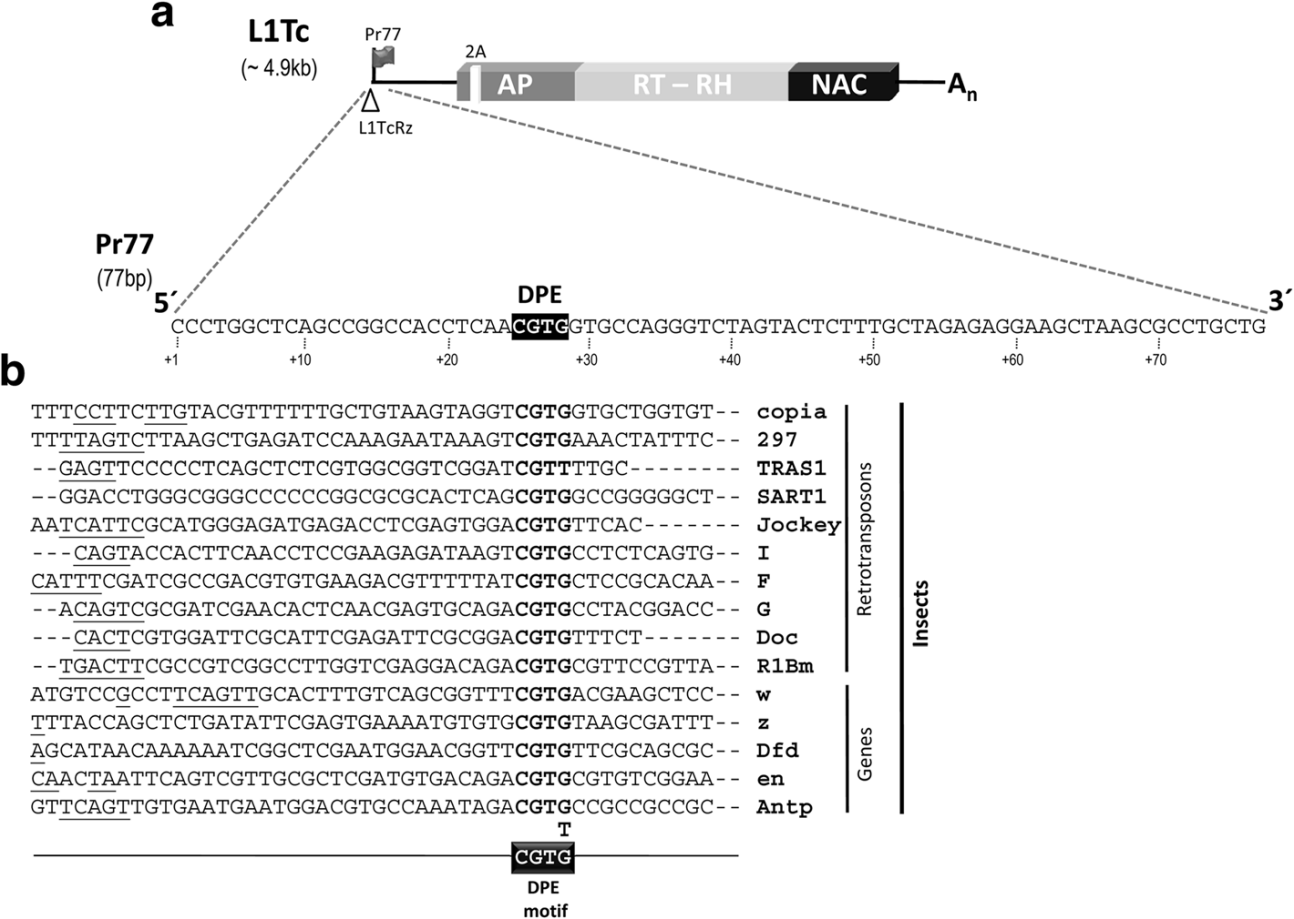
Diagram of the L1Tc element including the Pr77 sequence. **a** Diagram of the L1Tc retrotransposon from *T. cruzi*. The coding sequences for AP endonuclease (AP), reverse transcriptase (RT), RNaseH (RH), the nucleic acid chaperone (NAC) proteins, and a 2A-self cleaving sequence, are shown in *boxes*. The Pr77 internal promoter is represented by a *black flag*, and the L1TcRz ribozyme by an empty *arrow-head*. The sequence of the Pr77 promoter is shown and the DPE motif labelled in *bold face*. **b** Alignment of the nucleotide sequences of the 5′ region of the different types of insect retrotransposon and developmentally regulated genes. The transcription start sites are *underlined* when known. Nucleotides are numbered from the transcription start site position (+1). The DPE motif is indicated in *bold*. A diagram of the DPE motif consensus (CGTG) with the CGTT variant for the TRAS1 retroelement is shown below the alignment

In previous work, we reported the presence of an RNA pol II internal promoter involving the first 77 nucleotides (Pr77) of the 5′end of the L1Tc retrotransposon. Pr77-derived transcripts initiating at the +1 nucleotide of Pr77 are very abundant and readily translated [11]. To identify the sequences within Pr77 responsible for gene transcription activation, several sequence analyses were performed. No TATA box was found in the Pr77 upstream sequence, nor were any regulatory elements known to be involved in transcription predicted. However, comparison of the Pr77 sequence with *i*) LINEs from *Drosophila melanogaster* and other species with confirmed or expected RNA pol II internal promoters, *ii*) retrotransposons from *D. melanogaster*, *iii*) non-TATA genes from *Drosophila* and mammals, which are regulated and which have a transcriptionally important downstream element, and *iv*) some developmentally regulated non-TATA genes, revealed the maintenance in Pr77 of a small core sequence conserved in all the transcribed sequences described by Arkhipova et al. [26] (Fig. 1). This downstream promoter element is composed of 4 nucleotides, CGTG, and lies in Pr77 at positions +25 to +28 of the nucleotide +1 of L1Tc mRNA. This conserved sequence, and its location with respect to the reported transcription initiation site, corresponds to that described for downstream promoter elements known as DPEs [26]. When the Pr77 sequence comparisons were performed from different copies of L1Tc present in the genomes of Brener, BrenerEL, BrenerNEL, Dm28c, SylvioX10 and Marinkellei B7 strains (available at http://tritrypdb.org/), most of them were seen to be identical (laboratory data). Although some showed differences in one or two nucleotides, the complete DPE was conserved in more than 99 % of the elements (data not shown). This degree of conservation suggests this motif is essential in Pr77 promoter activity.

Analyses were then performed to determine whether the DPE of L1Tc was conserved in the Pr77 of other retrotransposons that show conserved sequences and 5′ end location. Sequence alignments were performed using CLUSTALW2 software and the Pr77 consensus sequences of other LINEs from Trypanosomes (such as L1Tc and *ingi*), their truncated versions (such as NARTc and RIME), SIDERs of African trypanosomes [9] and *Leishmania* species [27], and DIREs from trypanosomatids [6], which despite accumulating a large number of mutations maintain Pr77 at their 5′ ends. The DPE motif CGTG was also conserved in terms of sequence composition and distance to the TSS in the consensus sequence of L1Tco from *T. congolense*, the *ingi* elements of *T. brucei*, *T. congolense* and *T. vivax*, the elements corresponding to the truncated versions of the L1Tc- and the *ingi*-retrotransposons (NARTc and RIME) of *T. cruzi*, *T. brucei* and *T. vivax*, in SIDER 1 of *T. congolense* and SIDER1a of *T. vivax,* SIDER 2 from *T. brucei*, and SIDER 2A of *L. infantum*, *L. mexicana*, *L braziliensis, L. panamensis* and *L. major* (see Additional file 1: Figure S1). However, in the Pr77 sequence of SIDER1b-c of *T. vivax* and *T. brucei*, and that of SIDER 2B of *L. major*, the DPE motif was incomplete. The Pr77 DPE motif is also conserved in the consensus sequences described by Bringaud [6] in the DIRES elements of *T. cruzi* and *T. brucei* (Additional file 1: Figure S1).

### Pr77 promoter regions involved in transcriptional activity

**Fig. 2:**
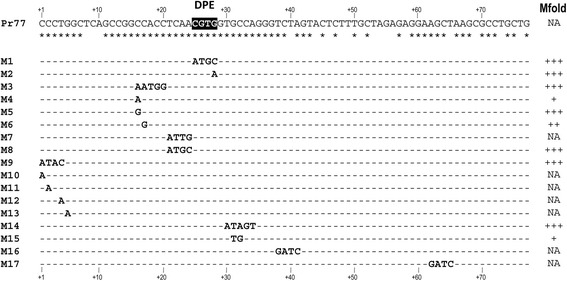
Point mutations in the Pr77 sequence in stable transfectants for analysis of the involvement of these nucleotide positions in the transcription capacity of the Pr77 promoter. The DPE motif is shadowed in the Pr77 L1Tc sequence. Nucleotides are numbered from the L1Tc transcription start site (+1). Conserved nucleotides among L1Tc and NARTc from *T. cruzi,* and *ingi* and RIME from *T. brucei*, are indicated by *asterisks* below the Pr77 sequence. Mutant names (from M1 to M17) are shown on the left hand side and the corresponding nucleotide substitution and position indicated for each mutant. Also on the left hand side it is indicated whether the DNA secondary structure is not affected (NA), slightly affected (+) or very affected (+++) by the point mutation, as predicted by the Mfold program

To determine whether the identified Pr77-DPE was involved in transcription, L1Tc-Pr77 was cloned upstream of the luciferase reporter gene into the pTEX transfection vector (pTEXPr77Luc). Different point mutations were introduced at 17 locations in the 77 nucleotide sequence (pTEXPr77Mut1-17Luc) (Fig. 2) to examine the participation of other conserved nucleotides that might also participate in Pr77 promoter activity.

Mutations were mainly introduced into the conserved nucleotides of the Pr77 sequences of L1Tc, NARTc, *ingi* and RIME (shown with an asterisk below the Pr77 sequence in Fig. 2). Thus, the DPE motif - the most likely candidate responsible for Pr77 promoter activity - was completely (mutant 1) or partially (mutant 2, position 28, GxA) substituted. The DPE upstream region (CCACC) that, along with the DPE motif, forms a stable structure (as predicted by the Mfold program) was completely mutated (mutant 3, positions 16–20, CCACC x AATGG) or partially point mutated (mutants 4, 5 and 6; position 16, CxA and CxG and position 17, CxG, respectively), as well as the nucleotides of the sequence located between these two sequences (mutants 7 and 8: positions 21–24, ATTG and ATGC x TCAA, respectively). The first nucleotides forming part of the L1Tc-Pr77 mRNA were also completely substituted (mutant 9, positions 1–4, ATAC x CCCT) or partially mutated (mutants 10–13: positions 1, 2 and 4, AxC, AxC and AxT, respectively). Other regions of Pr77, including conserved and non-conserved nucleotides were also mutated. Mfold predictions were made for all the sequences (Fig. 2). On the other hand, some of the described point mutations strongly compromised the stable secondary structure (+++) predicted for Pr77 L1Tc (mutants 1, 2, 3, 5, 8, 9, 14) while others midly (+ and ++) (mutants 4, 6 and 15,) (Fig. 2) or had no influence at all on the secondary structure (NA, mutants 7, 10–12, 16–17). Subsequently, *T. cruzi* epimastigotes were transfected with one each of these constructs (Pr77 wild type or Pr77-derived mutants 1–17) by electroporation, and stable transfectants generated by selection under G418 pressure.

**Fig. 3:**
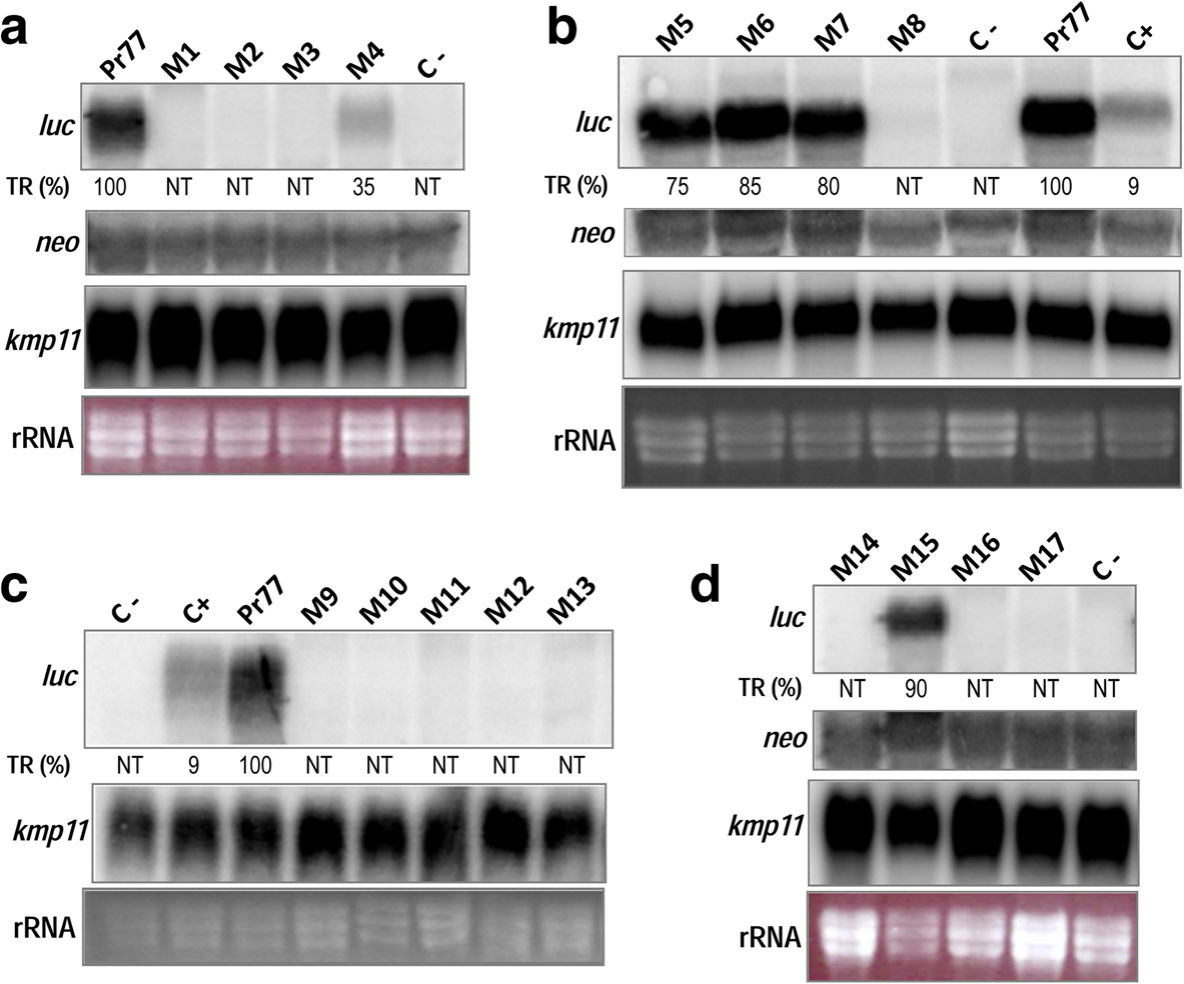
Analysis of transcriptional activity of Pr77-derived sequences in *T. cruzi* stable transfectants by northern blotting. **a**, **b**, **c**, **d** Cytoplasmic RNA from *T. cruzi* stable transfectants eletroporated with pTEXPr77Luc or pTEXPr77Mut1-17Luc, was electrophoresed in 1 % denaturing agarose gel, transferred to nylon membranes, and hybridized with ^32^P-labelled Luc *(luc)* and KMP11 *(kmp11)* coding sequences as probes. The ethidium bromide staining of ribosomal RNAs is shown below each panel (**a**-**d**). Cytoplasmic RNA from wild type Y epimastigotes and parasites transfected with the pTEXLuc construct were used as negative and positive controls (C- and C+ respectively). The northern blots were performed three times. The percentage of transcribed Luc mRNA when using the pTEXPr77Luc construct is shown below the *luc* hybridization panel as TR (%)

The promoter capacity of each Pr77-derived mutant was analyzed by northern blotting and quantification of the corresponding Pr77-derived cytoplasmic transcripts following hybridization with the α-^32^P dCTP *Luc* coding sequence used as probe. The variations in the episomal copy number, able to be transcribed, were analyzed in most transfectants by hybridization with the α-^32^P dCTP *neo* probe (Fig. 3 panels a-b and d) to detect the level of *neo* mRNA derived from the transfection vectors employed. Differences in RNA quantities were checked by staining the gels with ethidium bromide and filters normalized by hybridization with the α-^32^P dCTP *kmp11* probe (Fig. 3 panels a to d). As expected, abundant *Luc* mRNA (2100 nt approximately) was seen in the pTEXPr77Luc transfectants (lane Pr77 in Fig. 3a, b and c) - approximately 11 times that detected for the pTEXLuc transfected parasites (lane C+ in Fig. 3b and c). As shown in Fig. 3a-d, the DPE motif is essential for transcription activity since its complete (mutant 1) or partial modification (mutant 3) abolishes the promoter activity. Several additional nucleotides also appeared to be strongly involved in transcription, since their substitution abolished (mutants 3, 8–14, 16–17) or diminished (mutant 4) Pr77 promoter activity. However, the Pr77 mutants 5, 6, 7 (Fig. 3, panel b) and 15 (Fig. 3, panel d) maintained a transcriptional activity similar to that of the wild type construct. Since the level of *neo*^R^ and KMP11 mRNAs is similar in all the transfectants analyzed, the differences observed in the level of Luc transcripts are not a consequence of differences neither in the episomal plasmid load, able to be transcribed, nor variations in RNA quantities.

**Fig. 4:**
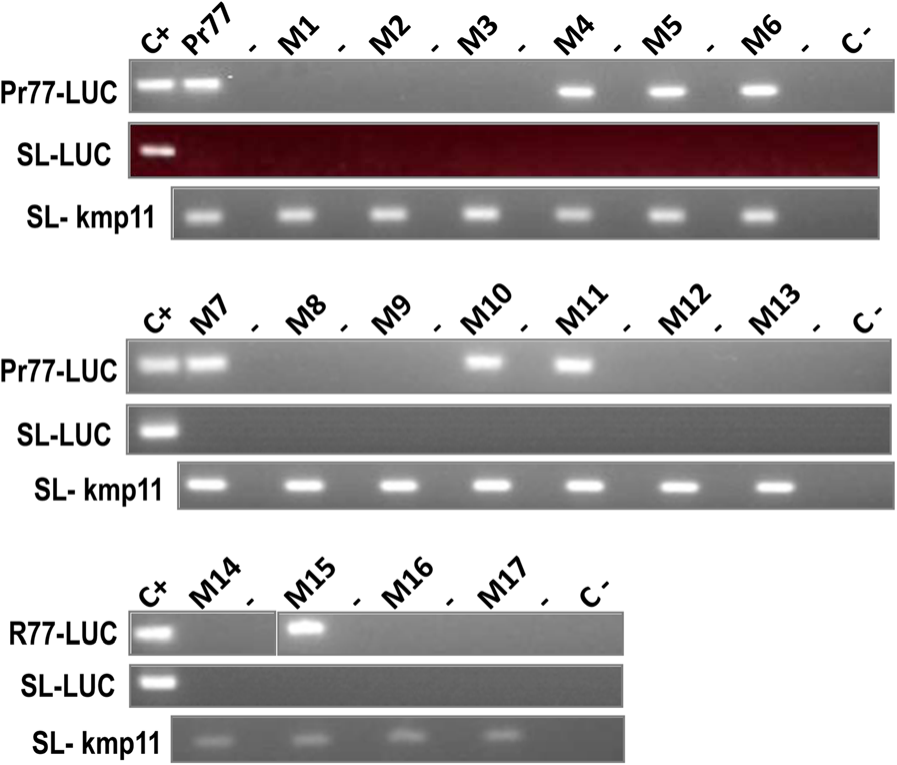
RT-PCR detection of LUC mRNAs in *T. cruzi* stable transfectants and of the 5′ end nature of Luc mRNAs. Cytoplasmic RNA from *T. cruzi* stable transfectants eletroporated with pTEXPr77Luc or pTEXPr77Mut1-17Luc were used as templates for reverse transcription and subsequent PCR amplification of the Luc mRNA 5′ end using a primer corresponding to the Pr77 5′ end (Pr77-LUC), or the SL primer (SL-LUC), as sense primers. As a control of RNA quality, the 5′ end of the *kmp11* RNA was reverse transcribed and PCR amplified. The pTEXPr77Luc and pGEMTSL-Luc plasmids were used as DNAs template in positive control reactions; no DNA was used in negative control reactions. Mutants are numbered from 1 to 17 (M1-17)

In a different attempt to detect possible low rates of Luc transcription in any Pr77-derived construct in the mutants 1–3, 8–14 and 16–17 transfectants, the luc messenger was reverse transcribed employing a Luc reverse primer (CTC7). The generated cDNA was then amplified by PCR using a sense primer corresponding to the 5′end of the Pr77 sequence (5′R77) plus the antisense Luc primer CTC8 (see Additional file 2: Figure S2a). As a quality control for the transfectant mRNAs, RT-PCR was performed to detect KMP11 mRNA as previously described [11]. The antisense kmp11 primer was used for reverse transcription and the spliced leader sense primer in subsequent PCR amplifications. These assays detected Pr77-derived transcripts in the parasites transfected with constructs 4–7 and 15. In addition, an amplification band was detected corresponding to the expression of the Luc messenger in the reactions for mutants 10 and 11 (Fig. 4). The data suggests that point mutations at positions 1 and 2 (mutants 10 and 11) strongly affected but do not abolished the transcription activity of Pr77 promoter as the Pr77-derived-Luc mRNAs from mutants 10 and 11 were detected by RT-PCR, a more sensitive technique that Northern blot. To determine whether any of the Pr77-dervied Luc transcripts had been processed by trans-splicing, the luc cDNA synthesized with the CTC7 primer was PCR-amplified with a primer corresponding to the spliced leader sequence and the CTC8 Luc antisense primer (see Additional file 2: Figure S2a). No bands were observed in any reaction with the exception of the plasmid DNA employed as a positive control (Fig. 4, SL-LUC panel). This was taken as an indication that that all Luc mRNAs derived from Pr77-mutated constructs lacked the spliced leader sequence at the 5′ end (Fig. 4, SL-LUC reactions). In contrast, and as expected, the reactions performed to analyze the 5′end of the kmp11 mRNAs showed these to be processed by trans-splicing (Fig. 4, SL-kmp11 reactions), and that the quality and integrity of each RNA sample was appropriate.

**Fig. 5:**
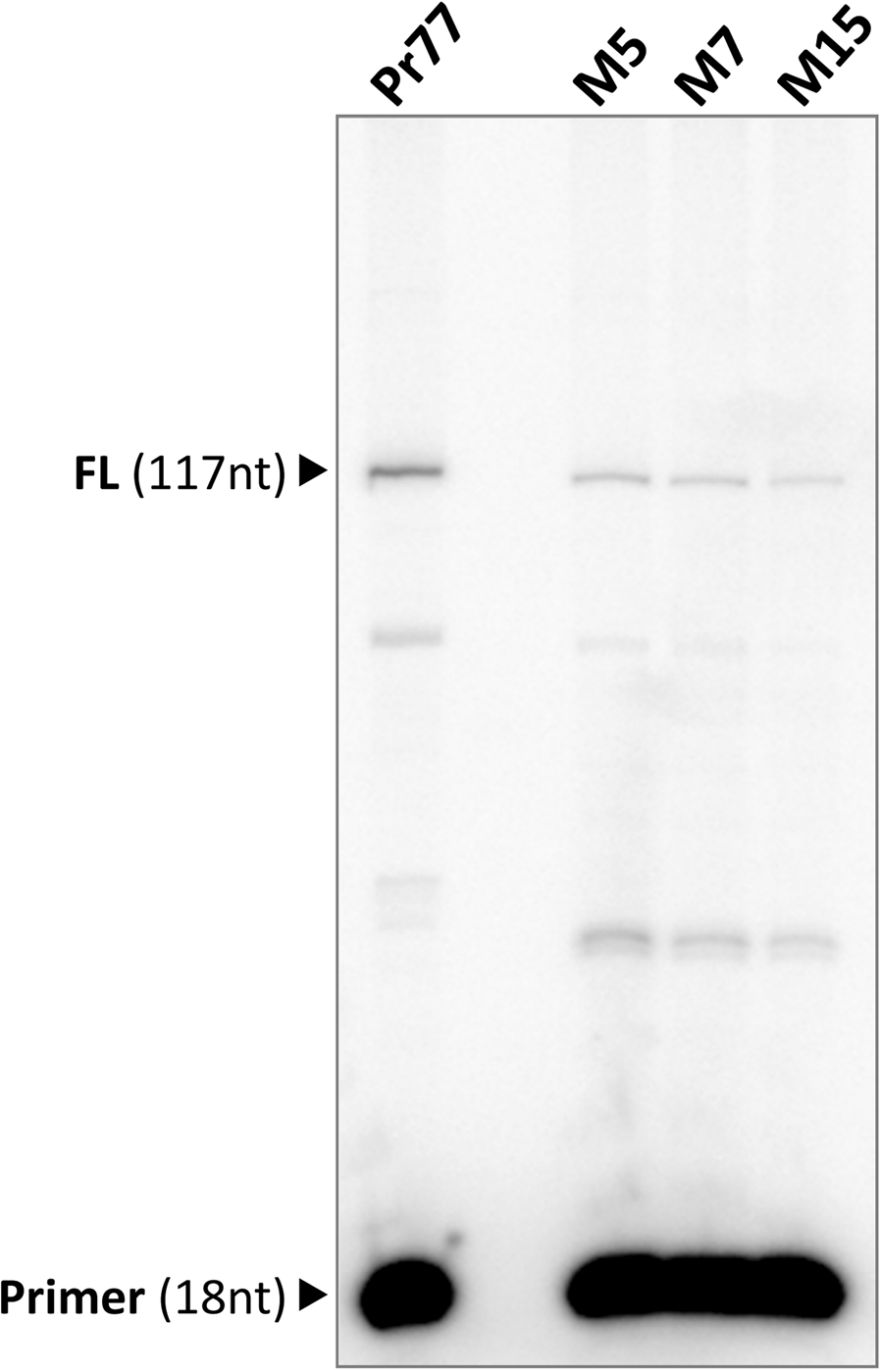
Determination of the *in vivo* transcription start site of Luc mRNA by primer extension. Total RNAs from *T. cruzi* transfected with the pTEXPr77Luc (Pr77), pTEXPr77M5Luc, pTEXPr77M7Luc or pTEXPr77M15Luc constructs (M5, M7 and M15) were used for primer extension of LUC mRNAs employing a γ-ATP^32^ Luc antisense primer (LUC28rev). The cDNA products were resolved in 8 M urea 8 % polyacrylamide gels. The full length extension products (117 nt in length), consistent with the TSS (FL) of Pr77 (nt +1) and 18-mer radiolabelled oligo LUC28rev, are indicated

To further determine the transcription initiation site of the Pr77-derived transcripts, and to identify differences between the mutant constructs in non-essential nucleotides and the wild type Pr77, primer extension was performed (Fig. 5). Total cytoplasmic RNAs from the Pr77 wild type Luc construct was used as positive control since Pr77-derived transcripts have been established to start at nucleotide +1 of Pr77 [11, 20] and three transcription-positive Pr77-derived mutant constructs (mutants 5, 7 and 15) were used as substrates in primer extension analysis involving the antisense luc primer (see Methods and scheme in Additional file 2: Figure S2b). A cDNA extension product of 117 nt was generated for both the wild type Pr77 (positive control) and all mutants. The length of this fragment corresponded to having been transcribed from the first nucleotide of the Pr77 sequence since an extension band of the same size than that generated in the parasites transfected with the Pr77 wild type Luc construct was detected. Thus, as for Pr77 [11], transcription mediated by mutants 5, 7 and 15 initiates at nucleotide +1, corroborating the idea that Pr77-derived mutant transcripts lack the splice leader sequence, as deduced from the RT-PCR data shown in Fig. 4 (SL-LUC reactions).

### Parasite nuclear proteins bind specifically to the Pr77 sequence and to the DPE itself

**Fig. 6:**
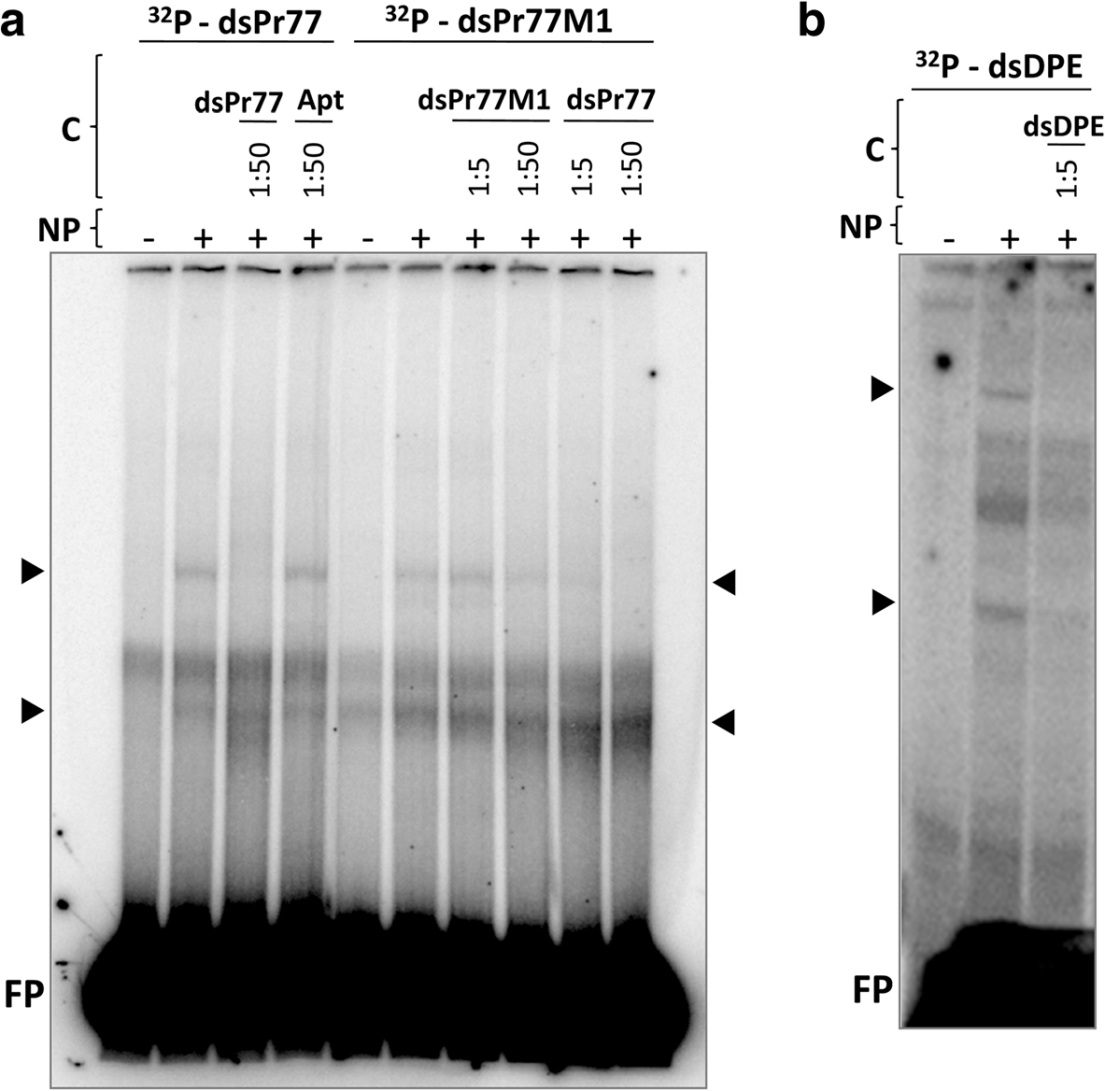
Binding of *T. cruzi* nuclear proteins to the Pr77, DPE-mutated Pr77, and DPE-bearing sequences. **a** Double-stranded DNA corresponding to the Pr77 (dsPr77), or Pr77 sequence mutated at the DPE motif (dsPr77M1), were 5′ end radiolabelled with γ-ATP^32^ and loaded onto native 6 % polyacrylamide gels with (+) or without (−) incubation with *T. cruzi* nuclear proteins (NP). Competition assays were performed by adding as competitors (C) non-labelled DNAs corresponding to the Pr77 sequence (dsPr77), a mixture of aptamers (dsApt) or dsPr77M1 to the reactions at a ratio of 1:50 or 1:5 as indicated in each panel. **b** Double-stranded DNA corresponding to the Pr77 sequence bearing the DPE motif (dsDPE, nucleotides 12 to 33 of the Pr77 sequence) was 5′ end radiolabelled with γ-ATP^32^ and loaded onto native 12 % polyacrylamide gels with incubation (+) or without (−) incubation with *T. cruzi* nuclear proteins (NP). Competition assays were performed by adding as competitor (C) non-labelled dsDPE DNA to the reactions at a ratio of 1:5. Shifted bands are indicated with an *arrowhead*, and the free probe indicated as ‘FP’

EMSAs were next performed to determine whether the nuclear proteins of *T. cruzi* specifically bind to the Pr77 sequence. A ^32^P-radiolabelled double-stranded fragment corresponding to the Pr77 full-length sequence (^32^P-dsPr77, Fig. 6a) was incubated with 3 μg of parasite nuclear protein extract, and the reaction resolved in 5 % polyacrylamide native gels. Two shifted bands were detected, indicating binding between the Pr77 sequence and the parasite proteins (Fig. 6a, lane +). No bands of reduced mobility were detected when binding assays were performed using a ^32^P-labelled probe corresponding to the human IL6 sequence and parasite nuclear extracts (data not shown). The shifted complex formed between the Pr77 sequence and parasite nuclear proteins (Fig. 6a, lane +) seems to be the result of specific binding between this particular sequence of DNA and the parasite nuclear proteins. Certainly, binding displacement between Pr77 and the nuclear proteins was entire when a 50-fold excess of unlabelled dsPr77 competitor DNA was added to the reaction (lane 1:50 dsPr77 in Fig. 6a). However, no displacement of the shifted complex was seen when a pool of 77 bp-long aptamer fragments with variable sequence compositions was included in the binding reaction as a competitor (1:50 dsApt in Fig. 6a).

The involvement of the DPE in the observed binding was examined via EMSAs performed with the nuclear protein extracts employed above and a ^32^P-labelled dsDNA corresponding to the Pr77 sequence of mutant 1, i.e., which had completely mutated DPE (see mutant 1 in Fig. 2). Lane ^32^P-dsPr77M1 of Fig. 6a shows a shifted band (+), the result of binding between the DPE-mutated Pr77 sequence and the parasite nuclear proteins. A partial displacement of the binding complex was seen when a 1:50 excess of cold DNA corresponding to the Pr77 sequence of mutant 1 was added to the reaction. When the competitor was cold DNA with the Pr77 sequence, a displacement of the DNA-protein complex was observed at a DNA/competitor ratio of 1:5. This displacement was entire when a 1:50 ratio was employed (Fig. 6, panel a). These findings suggest that parasite nuclear proteins specifically bind to different regions of the Pr77 sequence, although the strongest binding is with the DPE.

To corroborate the involvement of the DPE motif in the binding capacity of the nuclear proteins, an EMSA was performed using nuclear extracts of the parasite and a ^32^P-labelled DPE-containing a dsDNA fragment with a sequence corresponding to nucleotides +12 to +33 of the Pr77 sequence (^32^P-dsDPE). A band of reduced mobility was obtained when the total proteins of the parasite were incubated with the mentioned ^32^P-labelled dsDPE DNA (Fig. 6b). This binding was displaced by a 5-molar excess of cold dsDPE DNA, indicating sequence-specific binding.

### Independent of DNA secondary structure, parasite nuclear proteins bind to the sense strand of the sequence bearing the DPE

**Fig. 7:**
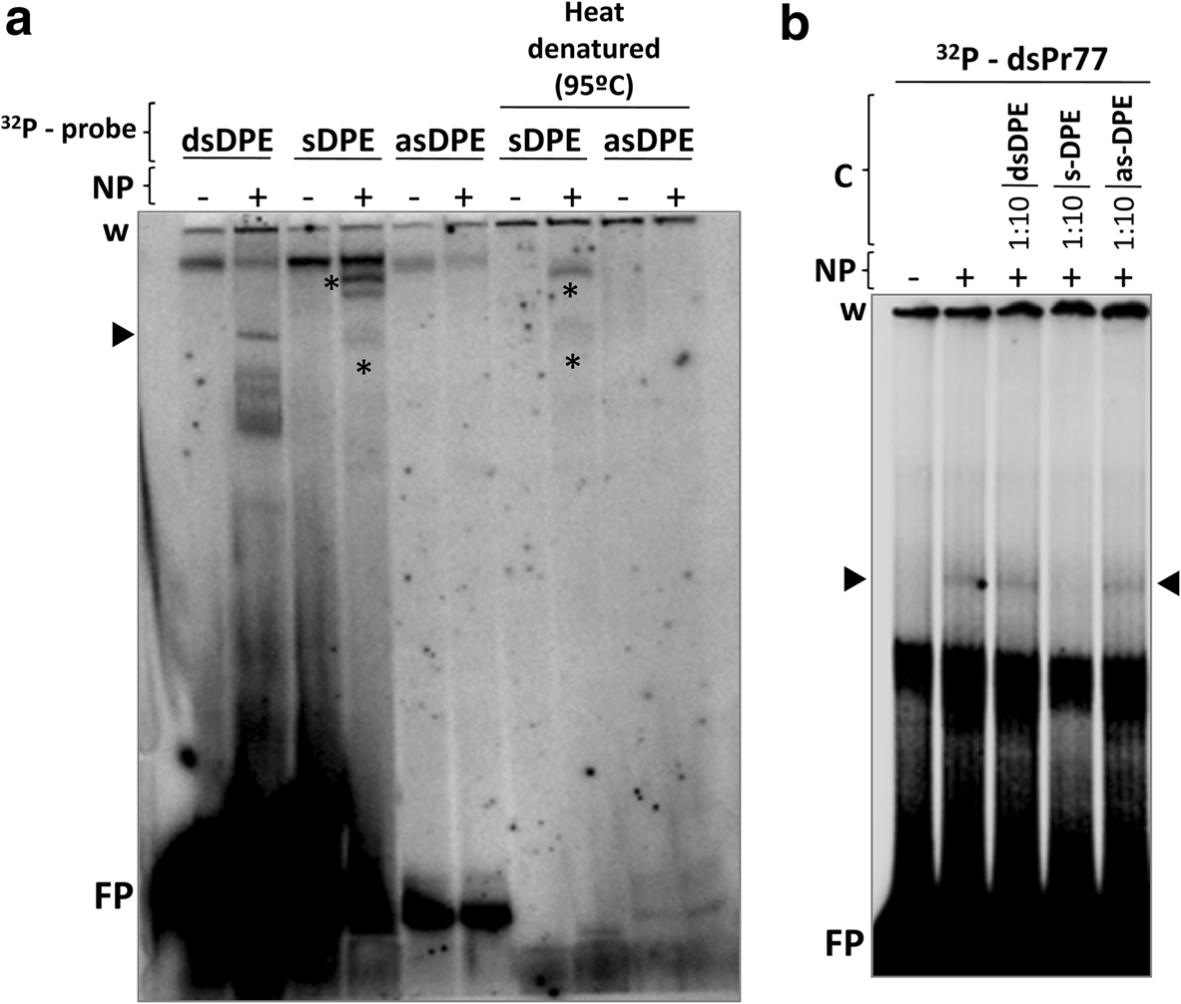
Binding analysis of *T. cruzi* nuclear proteins to DPE-bearing DNA as double strand DNA and single strand DPE-bearing oligos (sense and antisense) in native and heat denatured conditions. **a** DNA corresponding to the double-stranded DPE-bearing Pr77 sequence (dsDPE, nucleotides 12 to 33 of Pr77), single stranded sense DPE oligo (sDPE), single stranded antisense DPE oligo (asDPE) and the heat denatured sDPE and asDPE oligos, was 5′ end radiolabelled with γ-ATP^32^ and 80.000 cpm of each radiolabeled probe loaded onto native 12 % polyacrylamide gels with (+) or without (−) incubation with *T. cruzi* nuclear proteins (NP). The mobility of the highest amount of radiolabelled free probe is indicated as free Probe (FP). *Asterisks* indicate the shifted bands that are formed when nuclear proteins are incubated with both ^32^P- single stranded sense DPE and heat denatured ^32^P- single stranded sense DPE. **b** Binding of nuclear proteins to the Pr77 sequence and competition assays with DPE-bearing DNAs. Double-stranded DNA corresponding to the entire Pr77 sequence (dsPr77 probe) was 5′ end radiolabelled with γ-ATP^32^ and loaded onto native 6 % polyacrylamide gels with incubation (+) or without (−) incubation with *T. cruzi* nuclear proteins (NP). Competition reactions were performed by adding to the reaction non-labelled DNAs corresponding to the dsDPE sequence, the single stranded DPE oligo (sDPE) or the single stranded DPE antisense oligo (asDPE) as competitors (C) at ratios of 1:10. Shifted bands are indicated by *black arrows* on both sides of the gel (►). Gel wells are indicated as ‘*w*’

To determine whether the nuclear proteins bound the sense or antisense strand of the Pr77 DNA, binding reactions were performed incubating the ^32^P-labelled DPE in the form of a double-stranded DNA molecule (dsDPE, Fig. 7a), and the DPE oligos corresponding to the sense (sDPE of Fig. 7a) and antisense sequences (asDPE, Fig. 7a), with the parasite nuclear proteins. Specific, shifted bands were detected when the dsDPE DNA and sense DPE oligo were used (lane + in the dsDPE and sDPE groups of Fig. 7a), but not when the asDPE oligo was used (lane + in the asDPE panel of Fig. 7a). Together, these data suggest that, in DNA binding by *T. cruzi* nuclear proteins, the DPE motif is strongly involved with the sense single-stranded sequence, apparently being required in DNA-protein recognition.

The involvement of the Pr77 DNA secondary structure in the DNA binding capacity of the DPE-sequence by parasite nuclear proteins was examined by EMSAs performed using separately heat-denatured sense and antisense strands of the DPE oligo (sDPE and asDPE lanes respectively in Fig. 7a). Conformer formation was seen when the nuclear proteins were incubated with the sense DPE oligo and denatured sense DPE oligo (asterisks in lines + in ‘sDPE’and ‘sDPE heat denatured’ panel Fig. 7a) but not with the denatured antisense oligo (line + in ‘asDPE heat denatured’ panel Fig. 7a). This was taken as an indication of the sequence specific binding of the *T. cruzi* nuclear proteins to the DPE bearing sense sequence of 22 nucleotides in length which together with the DPE motif includes nucleotides that in mutants 3, 8 and 14 showed to compromise the transcription activity of the Pr77 sequence (Figs. 2 and 3). This was corroborated by binding assays involving *T. cruzi* nuclear proteins and the ^32^P-labelled-dsPr77 sequence, and binding displacement via the addition of cold double-stranded DPE oligos and the corresponding sense- and antisense DPE oligos. Thus, and as observed in Fig. 7b (lane sDPE), the DPE sense oligo displaced the conformer produced as a result of the binding of nuclear proteins to the dsPr77 sequence. This did not occur when using the same amount of asDPE oligo or 1:10 of the dsDPE cold DNA as competitors. Together, these data suggest that Pr77 has binding sites for nuclear proteins other than the DPE, although the latter, in the sense strand, is the site of strongest binding.

## Discussion

Transcription is the first step in the mobilization of retrotransposons and is critical for the successful maintenance of these elements in host genomes. Many non-specific sites for the insertion of non-LTR retrotransposons are transcribed from their 5′ ends by internal promoters [28, 29]. This gives transcriptional autonomy to these elements - they are independent of any regulatory sequences upstream of the insertion site.

The present work describes the identification of a downstream core promoter element in Pr77 of the L1Tc retrotransposon, known to drive gene transcription via RNApol II [11]. The core promoter is defined as the DNA stretch that directs the initiation of transcription by the latter enzyme. These core promoter elements are modular in structure and may contain elements such as a TATA box, a TFIIB recognition element (BRE), an Inr (initiator) element, a motif ten element (MTE), and a downstream promoter element (DPE) [30]. However there are no universal promoter elements. The Pr77 DPE motif is just four nucleotides in length (CGTG), located between positions +25 to +28 relative to the previously described TSS (+1 nucleotide of the Pr77 promoter). Following the Juven-Gershon, T. and Kadonaga, J.T. description [31] and in contrast to the “dispersed” promoters with TSSs, the presence of a DPE motif within the Pr77 sequence shows Pr77 promoter to be of the focused type. This is consistent with the results of previous studies on TSS-derived transcripts of Pr77 [11]. The Pr77 DPE is conserved in terms of sequence composition and location within the consensus sequences of the Pr77-hallmarks of trypanosomatid retrotransposons, independent of their coding or non-coding nature or degree of degeneration - with the exception of the TbSIDER1, TvSIDER1b-c and LmSIDER2b elements which all carry a mutated DPE. Experimental evidence of the functionality of the Pr77 sequences of these elements is still required.

The DPE in the Pr77 promoter sequence is the first consensus downstream promoter element to be identified in trypanosomatids. The DPE motif is conserved from *Drosophila* to humans, and is usually located (approximately) +28 to +32 nt downstream of the TSS. It is more abundant in TATA-less promoters, although some promoters contain both DPE and TATA motifs [32, 33]. In some cases, DPE works as an antagonist of TATA-boxes since stimuli that activate DPE-dependent transcription repress TATA-dependent transcription [34]. There are many cases of DPE motifs within non-LTR retrotransposon promoters in insects. Such is the case of the *Drosophila* jockey, Doc, G, I and F, and the *Bombyx mori* SART1 and TRAS1 non-LTR retrotransposons. All these house a DPE motif in their core promoters (Fig. 1b) [26, 34, 35]. In some, the transcription start site has been experimentally determined, and shown to lie at a constant distance from the DPE motif (+28 to +32 nt downstream of the TSS).

The total or partial mutation of the DPE of L1Tc completely abolished the transcription capacity of Pr77, indicating this sequence to be essential for Pr77 promoter activity. However, other positions in the Pr77 sequence are involved in its transcription activity. As deduced from the results provided by the transfectants containing the mutated Pr77-Luc constructs, the first nucleotides of Pr77 (where the Pr77 RNA begins its work) are critical for L1Tc-Pr77 transcription activity. However, the point mutations in Mutants 9–13, which were directed at the first nucleotides of the Pr77 sequence, had no influence on the secondary structure of Pr77 as predicted by the Mfold program. Other positions in Pr77 also appear to be important for transcription capacity, some of which do influence the secondary structure of Pr77. Mutants 7 and 8, which bore mutations in the same position (nucleotides 21–24 relative to the +1 TSS) are an interesting case in point. Mutant 7 conserves the promoter capacity and the secondary structure of the sequence, while mutant 8 has lost the promoter activity and the secondary structure of Pr77. The nucleotides experimentally shown to be essential in the transcription activity of the Pr77 sequence are likely targets for the binding of transcription factors that mediate Pr77 transcription function.

EMSAs performed with protein extract showed the nuclear proteins of the parasite to specifically bind to Pr77. The shifted bands resulting from binding were displaced when Pr77 cold DNA was used as a competitor, but not when the same amount of a commercial mix of aptamers was used (Fig. 6a). The observed binding was sequence specific, and although the strongest binding involved the DPE sequence, other nucleotides also bound the nuclear proteins. This explains the shifted band seen when the DPE-mutated Pr77 sequence (dsPr77M1) was incubated with the nuclear proteins, and why the shifted conformers were more efficiently displaced with smaller amounts of dsPr77 than ds Pr77M1 (Fig. 6a). The nuclear proteins that bind to the DPE sequence do so on the sense strand of the DNA (Fig. 7). The identification of the nuclear proteins that specifically bind to the DPE motif and Pr77 sequence is currently in progress, together with the analysis of the capacity to compete the protein binding of the mutants that compromise the Pr77-transcriptional activity.

The DPE motif is a TFIID complex recognition site [36]. Footprinting analyses performed with the internal promoter of the *Drosophila* jockey (joc) mobile element (a TATA-less, DPE-containing promoter) showed that eTFIID binds to the promoter in a region that extends from the vicinity of the RNA start site to about +30 to +35 nt downstream [32]. This is consistent with the results of previous studies with these TATA-less promoters, which suggested that efficient transcription from them did not require sequences upstream of the RNA start site in the location where a TATA box would normally be located [37–40]. In turn, this is consistent with the promoter activity that mediates L1Tc transcription being restricted to the Pr77 sequence.

Although core promoter elements share the common function of directing the initiation of transcription, they may have different transcriptional properties and mechanisms, a result of their particular core promoter elements. Thus, the basal transcription factors that transcribe TATA-dependant core promoters are not able to mediate the transcription of DPE-dependant promoters [41]. Moreover factors such as TBP, Mot1 and NC2 influence transcription from TATA-dependant promoters differently to DPE-dependant core promoters [42]. Core promoter diversity thus allows for a range of complex regulatory mechanisms by which eukaryotes can control gene expression.

Trypanosomatid retrotransposons have spread Pr77 throughout the genome of host organisms [22], thus distributing a tool for the regulation of gene expression. The transcriptional activity of the Pr77 sequence and the known accumulation of retroelements in the strand switch regions of trypanosomatids where the polycistronic transcription is initiated may be an indication of the influence of Pr77 in this process. Actually, the number of L1Tc elements in frame with the genes where these elements are inserted is one order of magnitude higher that those elements that are out frame as deduced from the analysis of the L1Tc elements available in the *T. cruzi* genomes (http://tritrypdb.org) (data not published). Moreover, considering all the available *T. cruzi* genomes, the number of the L1Tc elements in the divergent regions is 1.47 times higher than that from convergent strand switch regions (data not published). Histone variants related to the initiation of polycistronic transcription in *T. brucei* has been also detected close to the repetitive sequences located in the subtelomeric and centromeric regions [43]. However, as these regions are poorly annotated they have not been analyzed in detail [43]. Together with the promoter activity of the Pr77 sequence at the DNA level, HDV-ribozyme activity has been reported for the Pr77 sequence at the RNA level [20–22]. It remains unknown whether these functions are coupled. Mutations of DPE compromise the promoter activity of Pr77 DNA, and impair the HDV-ribozyme function of Pr77 RNAs (data not shown) since the P3 loop, which is required for the correct HDV-folding of Pr77 RNA [20–22] is affected. Other point mutations in Pr77 that do not compromise the Pr77 promoter function has influence on the HDV-ribozyme activity when the nucleotides involved in loop formation are affected (data not shown). Work is underway to confirm the above and to identify the factors that mediate Pr77 promoter activity.

## Conclusions

The first 77 nucleotides of the *T. cruzi* L1Tc retrotransposon, Pr77, are conserved in most retrotransposons of trypanosomatids and has been consequently named as Pr77-hallmark. Herein, it is described the identification and implication of a downstream promoter element (DPE) in the promoter function of L1Tc Pr77 to which nuclear factors are strongly and specifically bound. Conservation of DPE sequence is maintained in the Pr77 sequence of most retrotransposons of trypanosomatids independently of their autonomous or non-autonomous character and their degree of degeneration. Accumulation of retroelements in the strand switch regions of trypanosomatids where the polycistronic transcription is initiated suggests that these elements are involved in this process reinforcing the important role of retroelements in gene expression regulation of host genomes.

## Methods

### Transfection vectors for *T. cruzi*

The firefly luciferase coding gene (*Luc*) was excised from the pGEMT-LUC (Promega®) vector by BamHI digestion, blunt-ended by Klenow enzyme treatment, and then SalI digested for direct cloning into different vectors. To generate the pTEXPr77Luc vector, the above-mentioned *Luc* insert was directly cloned into the pTEX(p-)R77CAT construct [11] which was HindIII-digested, Klenow-treated and then digested by SalI. The *Luc* insert was also cloned into the pTEX vector after HindIII digestion, Klenow- treatment and digestion with SalI to generate the pTEXLuc transfection vector.

Seventeen Pr77 mutants were generated by three consecutive PCR runs using a pair of sense and antisense complementary primers (see primers sequences in Additional file 3: Table S1) bearing different point mutations (mutants 1–9 and 14–17), or by a single PCR run with a sense primer containing the mutation at the 5′end of the Pr77 sequence (mutants 10–13), in all cases using the pTEXPr77Luc vector as a template. For generating mutants 1–9 (pTEXPr77Mut1-9) and 14–17 (pTEXPr77Mut14-17), an initial PCR run (PCR1) was performed employing the sense mutant primers M1s-M9s or the sense mutant primers M14s-M17s together with the Luc627 antisense primer, and using the pTEXPr77Luc vector as a template. A second PCR run (PCR2) then involved the antisense mutant primers M1as-M9as or the antisense mutant primers M14as-M17as, together with the M13-20 universal primer, using the pTEXPr77Luc vector as template. A third PCR run (PCR3) was then performed using 2 μl of a mixture of the amplicons produced in PCR1 and PCR 2 with the universal primer M13-20 and the Luc627 antisense primer. The amplified products were EcoRI-digested and directly cloned into the pTEXPr77Luc vector pre-digested with the same enzyme, thus replacing the non-mutated Pr77Luc fragment for those mutated. For generating the construct pTEXPr77Mut10-13, a single PCR run was performed using a sense primer bearing the required mutation (the M10s-M13s primers) plus the Pr77-Mas antisense primer that maps to the end of the Pr77 sequence and contains a BamHI restriction site. The 103 bp-long amplified products generated were BamHI-digested and cloned directly into the pTEXPr77Luc vector pre-digested with the same enzyme.

All PCR reactions were performed using the Expand High Fidelity PCR System (Roche®). The thermomocycler settings were: 30 cycles of 30 s at 94 °C, 30 s at a suitable temperature for each pair of primers, and 45 s at 72 °C. DNA fragment ligations were performed using T4 DNA Ligase (Roche®). Correct cloning was confirmed in all cases by DNA sequencing.

### Epimastigote culture and transfection

*T. cruzi* epimastigotes (Y strain) were grown at 28 °C in liver infusion tryptone (LIT) supplemented with 10 % (*v/v*) heat-inactivated fetal bovine serum (Gibco®). DNA from pTEXPr77luc, pTEXLuc and the mutant constructions (pTEXPr77Mut1-17Luc) was purified using the Wizard® Plus Maxipreps Kit (Promega®). Transfection was performed by electroporation with 100 μg of each DNA as previously described [44]. Epimastigote cultures were also electroporated in the absence of DNA. Transfectants were selected by growth selection in the presence of 250 μg/ml of G418 (Gibco®). The transfectants were not cloned but used for further analyses.

### Purification of parasite RNA and northern blotting

*T. cruzi* cytoplasmic RNA from each transfectant was isolated from epimastigotes in the logarithmic phase of growth by parasite lysis and phenol extraction as previously described [44]. For Northern blot analysis, 10 μg of purified RNA were size-fractionated on 1 % agarose/formaldehyde gels, and then transferred to Z-probe nylon membranes (Bio-Rad®) in a 10× SSC solution. Hybridization was performed as previously described [45] but using PerfectHyb^TM^ Plus Hybridization Buffer (Sigma®). The *Luc* and *neo* DNA hybridization probes were generated by PCR amplification using the LucFw (5′ CGCCATTCTATCCTCTAGAGGAT 3′) and LucRT (5′ CTCCGATAAATAACGCGCCC 3′) primers or neoFw (5′ GATCGGCCATTGAACAAGAT 3′) and neoRv (5′ ATACTTTCTCGGCAGGAGCA 3′) oligos, respectively and the pTEXLuc vector as a DNA template. The *T. cruzi KMP11* coding sequence was also PCR-amplified using the K1Tc and K2Tc primers as previously described [45]. *Luc* and *kmp11* DNAs were radiolabelled using [α-^32^P]-dCTP (Perkin Elmer®) and the Random Primed Labelling System (Stratagene®). The hybridization products were visualized and quantified using a PhosphorImager (Thypoon, Pharmacia®). Quantification analyses were performed using ImageQuant software (Molecular Dynamics®).

### Analysis of the Luc mRNA 5′ ends by RT-PCR

The 5′ end of the *Luc* mRNAs in the different transfectants was analyzed by RT-PCR (See scheme in Additional file 2: Figure S2a). For cDNA synthesis, 1.5 μg of cytoplasmic RNA from each transfectant was reverse transcribed using the CTC7 (5′-CCAGCGGTTCCATCC-3′) primer and M-MuLV reverse transcriptase (Roche®), according to the manufacturer’s instructions (60 min at 37 °C). PCRs were performed using the Expand High Fidelity PCR System (Roche®) and 5 μl of the synthesized cDNA and the 5′Pr77 (5′-CCCTGGCTCAGCCGG-3′) and CTC8 primers (5′-CCTCTAGAGGATAGAATGGCG-3′) (30 cycles of 1 min at 94 °C, 1 min at 55 °C, and 1 min at 72 °C), or the SLTc (5′-CGCTATTATTGATACAGTTTCTG-3′) and CTC8 primers (30 cycles of 1 min at 94 °C, 1 min at 60 °C, and 1 min at 72 °C). The synthesis of cDNA from the KMP11 transcript was performed using primer K280, and PCR amplification performed using the SLTc and kmp2 primers as previously described [45]. A reaction with no reverse transcriptase was performed as a negative control.

### Primer extension assay

Ten micrograms of RNase-free, DNase I-treated cytoplasmic RNA from *T. cruzi* parasites, and from parasites transfected with pTEXLuc, pTEXPr77Luc, or pTEX Pr77Mut1-17Luc, were used as templates to extend the cDNA products using the LUC28rev primer (5′-CTTTCTTTATGTTTTTGG-3′) and avian myeloblastosis virus reverse transcriptase (Promega®) according to the manufacturer’s instructions. The LUC28rev primer was 5′ end-labelled with γ-P^32^ATP using T4 polynucleotide kinase. The reactions were resolved in an 8 M urea 8 % polyacrylamide denaturing gel. Products were visualized using a PhosphorImager device (Typhoon, Amersham Biosciences®).

### Nuclear extracts

To purify *T. cruzi* nuclear protein extracts, the method described by Jianpig Ye et al. was used [46]. Briefly, 2 × 10^8^ epimastigotes in the logarithmic phase of growth were harvested by centrifugation and washed twice in 1× PBS. Parasites were resuspended in 500 μl lysis buffer (50 mM KCl, 0.5 % NP-40, 25 mM HEPES pH 7.8, 125 μM DTT, 1 mM PMSF and protease inhibitors) and kept on ice for 4 min to break the cell membrane. The nuclei were collected by centrifugation for 5 min at 13.000 rpm, and washed in Washing buffer (50 mM KCl, 25 mM Hepes pH 7.8, 125 μM DTT, 1 mM PMSF and protease inhibitors). Nuclear proteins were obtained by resuspension of nuclear pellets in 100 μl of extraction buffer (500 mM KCl, 25 mM Hepes pH 7.8, 10 % glycerol, 125 μM DTT, 1 mM PMSF and protease inhibitors) and recovered by centrifugation for 5 min at 14.000 rpm; the supernatants were stored at −80 °C. The total protein concentration was determined using the Micro BCA Protein Assay Kit (Thermo Scientific) using BSA as a standard. Nuclear proteins quality was checked by SDS-polyacrilamide gel electrophoresis.

### Electrophoretic mobility shift assays

To generate the dsDNA probes for electrophoretic mobility shift assays (EMSA), three pairs of complementary oligonucleotides (s-Pr77/as-Pr77, s-M1/as-M1 and s-DPE/as-DPE) were annealed as described previously by Heras et al. [17] to produce the dsPr77, dsPr77M1 and dsDPE probes respectively. Annealing was performed by heating the primer mix at 95 °C for 5 min and slowly cooling to room temperature. Additional file 3: Table S2 shows a list of the primer sequences used. Subsequently, duplexes were 5′ end-radiolabelled using 40 μCi of γ-^32^PATP (Perkin Elmer®) and 20 U of T4 PNK enzyme (New England Biolabs®) in a final volume of 10 μl, and passing through a G-25 Sephadex column. Three micrograms of nuclear extract were incubated with each ^32^P-labelled probe (80.000 cpm) in 1× binding buffer (12 mM Hepes, 4 mM Tris–HCl pH 8, 1 mM EDTA pH8, 125 ng/μl BSA, 2 μg Poly[dI-dC], 0.5 mM DTT and 6 % glycerol) for 15 min at room temperature. Competition assays were performed with 5, 10 and 50-fold excess (ng) of non-labelled probes previously extracted from a 6 % native polyacrylamide gel. Radiolabelled probes without competitors - referred to as free probes (FPs) - were included in all experiments. The lack of FP in the heat denatured lines is most likely due to the change in migration of the probe due to denaturation. Reactions were resolved in 6 % *w/v* non-denaturing polyacrylamide gels by electrophoresis (10 mA, 4 °C for 2.5–3 h). After drying, the gels were exposed to X-ray film and the bands visualized using the Typhoon system (Amersham®).

### Prediction of DNA secondary structure

Predictions regarding the DNA secondary structure of the Pr77 sequence, and of the Pr77-derived mutants, were made using the Mfold program (available at http://mfold.rna.albany.edu/?q=mfold) [47].

### Ethics approval

Ethical approval was not required for this study.

### Availability of supporting data

All the supporting data is included within the article and its additional files.

## Additional files

Additional file 1: Figure S1. Alignment of the Pr77 sequence of trypanosomatid retrotransposons. a) Multiple alignment of the consensus sequence of Pr77 from *T. cruzi* L1Tc and its homologs L1Tco in *T. congolense*, *ingi* from *T. brucei* and *T. congolense*, the truncated versions *T. cruzi* NARTc*, T. brucei* RIME and *T. vivax* RIME, SIDER1 from *T. congolense, T. vivax* SIDER 1a*, T. brucei* SIDER2*,* SIDER2a from *Leishmania infantum, L. mexicana, L. braziliensis, L. panamensis* and *L. major*, SIDER1b and c from *T. vivax,* SIDER1 from *T. brucei*, and SIDER2b from *L. major*. b) Multiple alignment of Pr77 of L1Tc and the consensus sequence of the DIREs from *T. cruzi* and *T. brucei*. The DPE motif is shown with a black shadow when conserved. (PDF 167 kb) Additional file 2: Figure S2. Diagram of: a) RT-PCR for analysis of the composition of the Luc mRNA 5′ end. The CTC7 primer was employed for the reverse transcription and cDNA synthesis of Luc mRNA in stable *T. cruzi* transfectants. PCR amplification was carried out with 5′R77 primer or the SLTc primers. b) Primer extension analysis to detect the transcription initiation site of Luc mRNAs in stable transfectants. The LUC28rev primer was used to extend the Luc mRNA using the AMV reverse transcriptase enzyme. (PDF 288 kb) Additional file 3:
**Sequences of the primers synthesized to generate (Table S1) the Pr77 mutants (Pr77 M1-17) and of those employed for use as (Table S2) probes in binding analyses (performed with nuclear protein extracts of the parasite).** (PDF 387 kb)

## Abbreviations

DPE: Downstream core promoter element
TPRT: target-primed reverse transcription
LINE: long interspersed nucleotide element
SINE: short interspersed nucleotide element
DIRE: degenerate *ingi*/L1Tc-related element
SSR: strand switch region
TSS: transcription start site
Luc: luciferase reporter gene
