# Supplementary material for: The Trypanosomatid Pr77-hallmark contains a downstream core promoter element essential for transcription activity of the Trypanosoma cruzi L1Tc retrotransposon
Source: BMC Genomics. 2016 Feb 9;17:105. doi: 10.1186/s12864-016-2427-6 (PMC4748587; doi:10.1186/s12864-016-2427-6)
Supplement: Additional file 1: Figure S1. — Alignment of the Pr77 sequence of trypanosomatid retrotransposons. a) Multiple alignment of the consensus sequence of Pr77 from T. cruzi L1Tc and its homologs L1Tco in T. congolense, ingi from T. brucei and T. congolense, the truncated versions T. cruzi NARTc, T. brucei RIME and T. vivax RIME, SIDER1 from T. congolense, T. vivax SIDER 1a, T. brucei SIDER2, SIDER2a from Leishmania infantum, L. mexicana, L. braziliensis, L. panamensis and L. major, SIDER1b and c from T. vivax, SIDER1 from T. brucei, and SIDER2b from L. major. b) Multiple alignment of Pr77 of L1Tc and the consensus sequence of the DIREs from T. cruzi and T. brucei. The DPE motif is shown with a black shadow when conserved. (PDF 167 kb) [file 12864_2016_2427_MOESM1_ESM.pdf]

a

```
                                         DPE
L1Tc          CCCTGGCTCAGCCGG---CCACCTCAACGTGGTGCCAGGGTCTAG---TACTCT------TTGCTAGAG---------AGGAAGCTAAGCGCCTGCTG- 77
L1Tco         CTCTGGTGCAGCCGG---CCACCTCAACGTGGTGCCAGGGTCCAG---TACTCT------TCATTGGAG---------AGGAAGCTAAGTGCCAGCTAC 78
ingi          CCCTGGCGATGCCGG---CCACCTCAACGTGGTGCCAGGGTCCAG---TACCCCG-----TATCATCGG---------GGGAAGCCAAGAGCCAGCAGT 79
Tcoingi       CCCTGGTGACGCCGG---CCACCTCAACGTGGTGCCAGGGTCTAG---TACTCC------GTCCAG--G---------AGGAAGCCAAGCGCCCGCATT 76
Tvingi        CCCTGGTGACGCCGG---CCGCCTCAACGTGGTGCCAGGGTCTAG---TGCTCC------GCCCAG--G---------AGGAAGCCAAGCGCCCGCACT 76

NARTc         CCCTGGCTCAGCCGG---CCACCTCAACGTGGTGCCAGGGTCTAG---TACTCT------TTGCTAGAG---------AGGAAGCTAAGCGCCTGCTG- 77
RIME          CCCTGGCGATGCCGG---CCACCTCAACGTGGTGCCAGGGTCCAG---TGCCCCG-----TATCATTGG---------GGGAAGCCAAGAGCCAGCAGC 79
TvRIME        CCCTGGTGACGCCGG---CCGCCTCAACGTGGTGCCAGGGTCTAG---TGCTCC------GCCCAG--G---------AGGAAGCCAAGCGCCCGCACT 76

TcoSIDER1     CCCTGATGTTAGTGGTAAACGCCTCATCGTGGCGTCAGGGTCTAG---TACCT---AGAAATGCT-TA---TTTCACAGGGAAGCTAAGATTTACCACG 89
TvSIDER1a     CCTTGATGTAAGTGGTAAACGCCTCATCGTGGCATCAAGGTCTAG---TACCTG--CGAAATTCCTTGG-AGTTCGGAGGGAAGCTAAGTTTTACCACG 93
TbSIDER2      CCCTGGTAAGGATGG-ATCTGCCTCAACGTGGCGCCAGGGTCCAG---TACCAGAAGAGAAATCGACTG---------GGAAGCCAAA-TGTTCCATC- 84
LiSIDER2A     CCCTGATAACGGGG---GACACCTCAGCGTGGTATCAGGGTCCAGTACCCACTC------TCTCTGTGG---------GGAAGCCAAGCAGCCCCTATT 81
LmexSIDER2A   CCCTGATAACATGGG-TGACACCTCAGCGTGGTATCAGGATCCAGTATCCACTC------TCTCTGTGG---------GGAAGCCAAGCAGCCCCTATT 83
LbraSIDER2A   CCCTGATGACGCGGA-AAGGTCCT-AGCGTGGTATCAGGGCCCG----CCCCCC------GCTCGGCGG---------GGAGGTCAGGCAGCCCCCTAT 78
LpanSIDER2A   CCCTGATGACGAGGA-AAGGTCCT-AGCGTGGTATCAGGGCCCG----CCCCCC------GCTCGGCGG---------GGAGGCCAGGCAGCCCCCTAT 78
LmSIDER2a     CCCTGATGACGAGG---GACACCTCAGCGTGGTATCAGGGTCCAG-TACACCCC------ACTCTGTGA---------GGAAGCCGAGCAGCTCCCTC- 79

TvSIDER1b     CCTTGATGTAGGTGGTAAACGCCTCATCGCGGGGTCAAGGTCTAG---TACCTG--CGAAATTCTTTGG-AGTTCGAAGGGAAGCTAAGTTTTGCCACG 93
TvSIDER1c     CCTTGATGTAAATGGCAAACGCCTCGTTGCGGCACCGTGGAATAG---AACCTA--CGAAGTTCATTGG-ATTTCTTAGGGAAGCGGAGTTTTACCACA 93
TbSIDER1      CCCTGACGTTAGTGGTAGCAGCCTCATTGTGGTGTCAGGGTCTAG---TACCCA--GGAAGAAT-TAAAATTTTCTGCGG-AAGCTAACTGTTACCACA 92
LmSIDER2b     CCCTGCCAATGCCG--AACCACTTCTG-GTGGTGACAGGGTCCAG---TGCCT------ACTACGTAGG---------GGAGGTCAGAGCGATGCATC- 77
              *  **     .  *      * *    * ** . *. *. . .      .                           .* .. * ..        :
```

b

```
                                        DPE
L1Tc          CCCTGGCTCAGCCGGCCACCTCAACGTGGTGCCAGGGTCTAGTACTCTTTGCTAGAGAGG-AAGCTA--AGCGCCTGCTG 77
TcDIRE1       CAGTGGCAATACCGGCCGCCCCAGCGTGGTGCCAGGGTGAATTGCTCCGTATTGTCGGGGGAAGCCAGCAATGCTTCCC- 79 Tc507229
              *. ****:.:.******.** **.************* :* *.***  *. *. .*.** **** *  *. ** * *


ingi          CCCTGGCGATGCCGGCCACCTCAACGTGGTGCCAGGGTCCAGTACCCCGTATCATCGGGGGAAGCCAAGAGCCAGCAGT--- 79
TbDIRE2       CCCTGGCGATGCCGGCCACCTCAACGTGGTGCCAGGGTCCAGTACCCCGTATCATCGGGGGAAGCCAAGAGCCAGCAGCGTT 82 Tb1a
TbDIRE1       CCCTGGTGATGCTGGCCACCTCATCGTGGTGCCAGGGTCCAGTACCTCGCCTATGCGTGGGAAGCCAAGAGCCGGCAAC--- 79 Tb1b
TbDIRE3       CCCTGCTGACGCCGGACACATTATCGTGATGCCAGGGTCTAGTACC-CGTCTAGTCTGTGGAAGCCAAGTGTCCGCATCATA 81 Tb5.204a
Tb9.146a      CCCTTGTGACGGCGGTCACCTCATCGTGGTGCAAGGGTCTAGTACTCCGTCCAG---GAGGAAGTCAAGTGCCCGCATTACT 79 TbDIRE2
Tb5.204b      ACATTGTGATTCCGACCACCTCATCGTGGTGCGAGGGTGTAGTACTCCGTCCAG---AAGGAATCCAACTGCCCCTATCACA 79 TbDIRE3
Tb2.19        CCCTGGTGAAGCCGGTCACCTCATCGTTGCGCCAGGGTCTAGTACTCTATCCAG---AAAGAAGCCAAGTGCCCTCATTAAA 79 TbDIRE2
Tb9.145a      TGTGGGTGACACCGGCTGATCCATCGTGGTACCAGTGTCTAATACTCCATCCAG---GAGGAAGCCAAGTGCCAGCATCAAA 79 TbDIRE3
Tb9.231a      TTGCTCTGAGTATGGCTGCCTCATCGTGGTTTCAGGGTCCAGTACCGCAAACAA---GAGGAAGACAAGTGCCCACATCGTA 79 TbDIRE2
                   **    *.  ..   *:*** .    ** **  *.***   . . .      .***  *** :* *   *
```

Additional file 1
