# Supplementary figures and images for: The Trypanosomatid Pr77-hallmark contains a downstream core promoter element essential for transcription activity of the Trypanosoma cruzi L1Tc retrotransposon
Source: BMC Genomics. 2016 Feb 9;17:105. doi: 10.1186/s12864-016-2427-6 (PMC4748587; doi:10.1186/s12864-016-2427-6)

**a**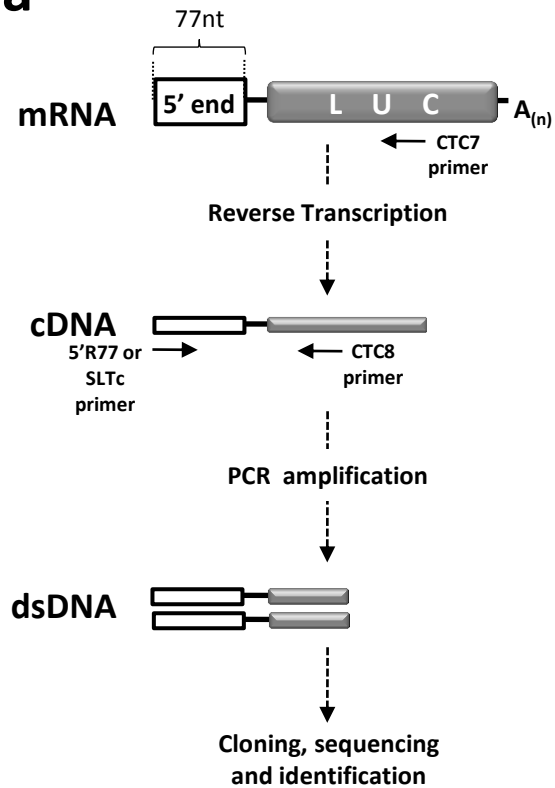**b**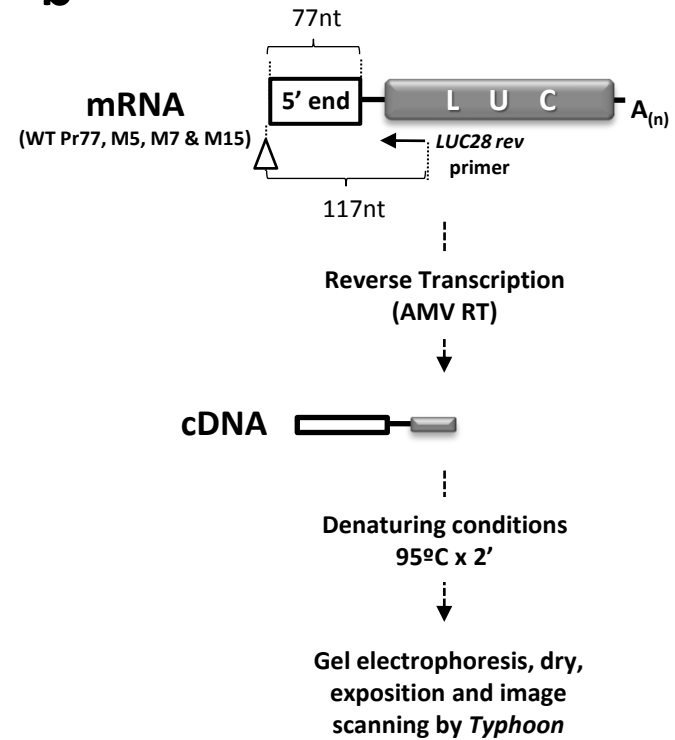

Supplement: Additional file 2: Figure S2. — Diagram of: a) RT-PCR for analysis of the composition of the Luc mRNA 5′ end. The CTC7 primer was employed for the reverse transcription and cDNA synthesis of Luc mRNA in stable T. cruzi transfectants. PCR amplification was carried out with 5′R77 primer or the SLTc primers. b) Primer extension analysis to detect the transcription initiation site of Luc mRNAs in stable transfectants. The LUC28rev primer was used to extend the Luc mRNA using the AMV reverse transcriptase enzyme. (PDF 288 kb) [file 12864_2016_2427_MOESM2_ESM.pdf]
