# Supplementary material for: The Trypanosomatid Pr77-hallmark contains a downstream core promoter element essential for transcription activity of the Trypanosoma cruzi L1Tc retrotransposon
Source: BMC Genomics. 2016 Feb 9;17:105. doi: 10.1186/s12864-016-2427-6 (PMC4748587; doi:10.1186/s12864-016-2427-6)
Supplement: Additional file 3: — Sequences of the primers synthesized to generate (Table S1) the Pr77 mutants (Pr77 M1-17) and of those employed for use as (Table S2) probes in binding analyses (performed with nuclear protein extracts of the parasite). (PDF 387 kb) [file 12864_2016_2427_MOESM3_ESM.pdf]

Table S1. The nucleotide sequence of the primers used to clone Pr77-derived promoter mutant construct

MUTANT No.	PRIMER	PRIMER SEQUENCE (5’→3’)
**M1**	M1s<br>M1as	5’-GGCCACCTCAA**ATGC**GTGCCAGGGTCTAG-3’<br>*5’-GACCCTGGCAC**GCAT**TTGAGGTGGCCG-3’*
**M2**	M2s<br>M2as	5’-CCACCTCAACGT**A**GTGCCAGGGTC-3’<br>*5’-GACCCTGGCAC**T**ACGTTGAGGTGG-3’*
**M3**	M3s<br>M3as	5’-GCTCAGCCGG**AATGG**TCAACGTGGTGC-3’<br>*5’-CCACGTTGA**CCATT**CCGGCTGAGCCAG-3’*
**M4**	M4s<br>M4as	5’-GCTCAGCCGG**A**CACCTCAACGTG-3’<br>*5’-CACGTTGAGG**T**GTCCGGCTGAGC-3’*
**M5**	M5s<br>M5as	5’-GGCTCAGCCGG**G**CACCTCAACGTGG-3’<br>*5’-CCACGTTGAGGTGCCCGGCTGAGCCAGG-3’*
**M6**	M6s<br>M6as	5’-GCTCAGCCGGC**G**ACCTCAACGTGG-3’<br>*5’-CCACGTTGAGG**T**CGCCGGCTGAGC-3’*
**M7**	M7s<br>M7as	5’-GCCGGCCACC**ATTG**CGTGGTGCCAGGG-3’<br>*5’-CCCTGGCACCACG**CAAT**GGTGGCCGGC-3’*
**M8**	M8s<br>M8as	5’-ACC**ATGC**CGTGGTGCCAGGGTC-3’<br>*5’-ACG**GCAT**GGTGGCCGGCTGAGC-3’*
**M9**	M9s<br>M9as	5’-AATTCGATAT**ATAC**GGCTCAGCCGGC-3’<br>*5’-GCTGAGCC**GTAT**ATATCGAATTC-3’*
**M10**	M10s<br>M10as	5’-CATTGGATCC**A**CCTGGCTCAGCCG-3’
**M11**	M11s<br>M11as	5’-CATTGGATCCC**A**CTGGCTCAGCCG-3’
**M12**	M12s<br>M12as	5’-CATAGGATCCCCC**A**GGCTCAGCCG-3’
**M13**	M13s<br>M13as	5’-CAATGGATCCCCCT**A**GCTCAGCCGGCC-3’
**M14**	M14s<br>M14as	5’-CAACGTGGT**ATAGT**GGTCTAGTACTC-3’<br>*5’-GTACTAGACC**ACTAT**ACCACGTTGAGG-3’*
**M15**	M15s<br>M15as	5’-CTCAACGTGGT**TG**CAGGGTCTAGTAC-3’<br>*5’-GTACTAGACCCTG**CA**ACCACGTTGAG-3’*
**M16**	M16s<br>M16as	5’-CTCAACGTGGTG**A**CAGGGTCTAGTAC-3’<br>*5’-GTACTAGACCC**T**GTCACCACGTTGAG-3’*
**M17**	M17s<br>M17as	5’-GGTGCCAGGG**GATC**GTACTCTTTGC-3’<br>*5’-GCAAAGAGTAC**GATC**CCCTGGCACC-3’*
**M18**	M18s<br>M18as	5’-GCTAGAGAGGA**GATC**AAGCGCCTGCTG-3’<br>*5’-GCAGGCGCTT**GATCT**CCTCTCTAGCAAAG-3’*
**FLANKING PRIMERS**	M13-20<br>Luc627<br>Pr77Mas	5’-GTAAAACGACGGCCAGT-3’<br>*5’-GGCCACACCCTTAGGTAACCCAG-3’*<br>*5 -CATTTGGATCCGCTTCAGCAGG-3*

Mutations nucleotides are indicated in bold; antisense primers are italicized.

Additional file 3

Table S2. The nucleotide sequence of the oligonucleotides used as probes and competitors in EMSA assays

OLIGONUCLEOTIDE	PRIMER SEQUENCE (5'→3')
s-Pr77	5'-CCCTGGCTCAGCCGGCCACCTCAA**CGTG**GTGCCAGGGTCTAGTACTCTTTGCTAGAGAGGAAGCTAAGCGCCTGCTG-3'
as-Pr77	5'-CAGCAGGCGCTTAGCTTCCTCTCTAGCAAAGAGTACTAGACCCTGGCAC**CACG**TTGAGGTGGCCGGCTGAGCCAGGG-3'
s-M1	5'-CCCTGGCTCAGCCGGCCACCTCAA**<u>ATGC</u>**GTGCCAGGGTCTAGTACTCTTTGCTAGAGAGGAAGCTAAGCGCCTGCTG-3'
as-M1	5'-CAGCAGGCGCTTAGCTTCCTCTCTAGCAAAGAGTACTAGACCCTGGCA**CGCAT**TTGAGGTGGCCGGCTGAGCCAGGG-3'
s-DPE	5'-ACCGGCCACCTCAA**CGTG**GTGCC-3'
as-DPE	5'-AGGCAC**CACG**TTGAGGTGGCCGG-3'
Apt	pool of variable sequence fragments of 77bp (aptamer selection) used as non-specific competitor

DPE motif is in bold face; Mutation of the DPE motif is indicated in bold and underlined

Additional file 3
